# Androgen receptor status predicts response to chemotherapy, not risk of breast cancer in Indian women

**DOI:** 10.1186/1477-7819-8-64

**Published:** 2010-08-04

**Authors:** Chintamani, Pranjal Kulshreshtha, Anurupa Chakraborty, LC Singh, Ashwani K Mishra, Dinesh Bhatnagar, Sunita Saxena

**Affiliations:** 1 Department of Surgery, Indian Council Of Medical Research, New Delhi, India; 2 Vardhman Mahavir Medical College, Safdarjung Hospital, New Delhi, 110023, India; 3 Institute Of Pathology, Indian Council Of Medical Research, New Delhi, India

## Abstract

**Background:**

Considerably little is known about the biological role and clinical significance of androgen receptor expression in breast cancer. The objectives of this study were to characterize *AR*-CAG repeat genotypes in a cohort of women with breast cancer and to determine the influence of AR on response to neoadjuvant chemotherapy and clinical outcome.

**Materials and methods:**

Genotyping of the *AR *CAG repeat region was done on 70 patients and 80 healthy aged- matched female controls. To assess response to NACT, tissue samples from 30 LABC cases were evaluated quantitatively by real time for AR mRNA expression. The clinical response was correlated with both the pre and post chemotherapy AR expression. The CAG alleles did not show differences between cases and controls when the mean of short, long and average length of both CAG alleles was considered. However, analysis when done defining short allele as CAGn < 20 (AR1) and the long as CAGn ≥ 20 (AR2), risk was found associated with AR2 allele with marginal significance (P = 0.09). Stratification by age of onset, FH, stage, grade ER and AR status failed to reveal any association with breast cancer risk. Genotype carriers with ≥20 CAGn showed decrease of AR mRNA expression although significance could not be established (P = 0.47). Tumours in responders had the higher AR mRNA expression levels in pre neo-adjuvant chemotherapy condition (p < 0.02) which got reduced after neoadjuvant chemotherapy and the difference was found to be significant (P = 0.014).

**Conclusions:**

Although, expansion of the CAGn in the *AR *gene doesn't show any major effect on breast cancer risk, patients with positive AR expression, pre neoadjuvant chemotherapy, were found to be good responders and a decrease in mRNA level of *AR *gene related to the chemotherapy-induced apoptosis could serve as an important independent predictor of response to NACT.

## Introduction

Breast cancer is one of the most frequent malignancies amongst women across the world, as well as in India [1]. In India, an average of 100,000 women is diagnosed with carcinoma of the breast and 40,000 women die of the disease every year [2]. Although breast cancer is currently the second most common cancer among Indian women (19%) after cervical cancer (30%), in the urban cancer registries of Delhi and Mumbai it has rapidly overtaken cervical cancer in frequency. In India majority of breast cancer cases (30-50%) present at locally advanced stage [3] managed by neoadjuvant chemotherapy (NACT) in surplus of surgery for both local and systemic control. Since endogenous steroid hormones (Estrogen, Progestron and Androgen) exposure is known to influence breast cancer risk, genes responsive to such hormones are currently being considered as plausible candidates for low-risk breast cancer genes.

The importance of estrogen-mediated and progesterone-mediated responses for normal mammary growth and development and during mammary carcinogenesis is well recognized (Anderson and Clarke 2004). Results from recent clinical trials with aromatase inhibitors, agents that suppress estrogen synthesis through peripheral aromatization, in postmenopausal women with ER- or progesterone-receptor-positive breast cancer reinforce the importance of estrogen in breast-cancer growth. Several large, randomized trials have compared aromatase inhibitors with tamoxifen in postmenopausal women with early or advanced steroid-receptor-positive breast cancer (Yager and Davidson 2006). Considerably little is known about the biological role and clinical significance of androgen and its receptor (AR) expression in breast cancer[4,5]. AR protein, functions as a transcription factor that regulate the transactivation of hormone responsive genes and is thus of specific interest. The exon 1 of AR gene contains trinucleotide repeat polymorphism, CAG (encoding for polyglutamine) which flank the N-terminal domain of the AR protein, where the transactivation activity resides. Remarkably, a CAG trinucleotide repeat is also a target for multiple RNA binding proteins which have functional impact on AR protein function [6,7]. Sparse epidemiologic data suggest that a long AR-CAG repeat yielding a less active AR may be associated with increased risk of breast cancer [6,7]. Polymorphisms in AR-CAG repeat have been intensively studied as determinant of susceptibility to prostate cancer in Indian population [8,9] however; its association with breast carcinoma in Indian population is not yet explored.

Further, it has been shown that AR positive breast cancer patients have prolonged survival and a better response to hormone treatment than AR negative patients [10]. It is believed that knowledge of the receptor status of all three receptors (ER, PR, AR) may identify more accurately those patients with breast cancer who are most likely to respond to endocrine treatment (Brentani 1986, Langer 1990, Kuenen-Boumeester 1992, Isola 1993, Collett 1996). Consistent with a role for AR in breast cancer outcome, AR potently inhibited ERα transactivation activity and 17β-estradiol-stimulated growth of breast cancer cells. Transfection of MDA-MB-231 breast cancer cells with either functionally impaired AR variants or the DNA-binding domain of the AR indicated that the latter is both necessary and sufficient for inhibition of ERα signalling. By binding to a subset of EREs, the AR can prevent activation of target genes that mediate the stimulatory effects of 17β-estradiol on breast cancer cells (Amelia 2009). AR can be activated in a ligand-independent manner by a number of growth factors including epidermal growth factor (EGF). Data on the importance of the interaction between polypeptide growth factors like EGF and the ErbB network of receptors with the AR in favour of cancer survival are now rapidly emerging [10,11]. Hence, the present study was undertaken to investigate the influence of CAG repeat length and its association with Breast Cancer risk in North Indian women. The study also evaluates the potential of androgen receptors as predictive markers for response to Neo-adjuvant Chemotherapy in locally advanced breast cancer. The study had the approval of the institutional review board and the ethical committee.

## Materials and methods

### Study population

In the present study, Seventy (70) histologically confirmed breast cancer patients referred to Institute of Pathology during January 2000 to December 2003 from the department(s) of Surgery and Cancer Surgery of Safdarjung Hospital, New Delhi, India were included. Initially 160 cases were selected for the study but a good number of cases were excluded due to insufficient histological and clinical information, patients not agreed to participate and unavailability of lymphocyte DNA. Selections of the patients were mainly based on following criteria: any breast cancer patient histologically confirmed and without any previous treatment; any breast cancer patient without any other malignancy. During the same time period, eighty (80) age-matched healthy women (±2 years) were selected as control group. Blood were collected from the women attending antenatal check-ups and blood bank donors in Safdarjang Hospital New Delhi. Mean age of patients was 40.9 years (SD ± 10.7) and controls were 39.3 years (SD ± 11.9 years). Among patients 50 (71.4%) cases were of early onset (≤40 years), 20 (28.5%) were of late onset and 11(15.7%) cases had family history of breast/ovarian cancer. Histopathology examination showed Infiltrating Duct Carcinoma in 74.2% cases and infiltrating lobular carcinoma in 7% cases. Twenty three patients presented with stage I and IIa, 39 patients with stage IIb & III (locally advanced) and 8 with stage IV. Out of 70 cases, eighteen were high grade tumours (III). Informed consent was obtained from all participating patients and the study was carried out with the approval of Ethical Review Committee of Safdarjung Hospital, New Delhi.

### Genotyping of AR-CAG repeats polymorphism

Peripheral blood samples (ca.10 ml) were collected into EDTA vials and genomic DNA was extracted from peripheral blood lymphocytes using standard phenol-chloroform extraction method. These genomic DNA were then used for genotyping of CAG repeat polymorphism in AR gene. An approximate 288 bp fragment was amplified using forward primer 5'- TCCAGAATCTGTTCCAGAGCGTGC-3' labeled with ABI-FAM (Applied Biosystems) and reverse primer 5'-GCTGTGAAGGTTGCTGTTCCTCAT-3'. Fluorescent amplified DNA along with LIZ standard and formamide were heat denatured at 95°C for 5 min., chilled on ice and loaded on 3130xl sequencer. Raw data was analyzed using ABI Gene Mapper software package.

### Hormone Receptor Status Analysis

Estrogen receptor (ER) status was estimated immuno-histochemically in 70 cases; 51 (72.8%) ER negative and 19 (27.2%) ER positive cases were included in the present study to find out the association between estrogen receptor status and androgen receptor AR2 allele genotype.

### Total RNA extraction from breast tissue and Quantitative Real-time RT-PCR

Total RNA was extracted from 40 (57.1%) histologically confirmed breast tumor biopsies using TRIzol reagent (Invitrogen, CA, USA) in accordance with the manufacturer's instructions. The quality of the RNA samples was determined by electrophoresis with a 1.5% denaturing agarose gels and staining with ethidium bromide and the 18 S and 28 S RNA bands were visualized under UV light and quantitated by Nano-dropspectrophotometrically (NanodropR ND-1000 UV-Vis Spectrophotometer (Nanodrop Technologies, Rockland, USA). RNA was reverse transcribed using high capacity cDNA archive kit (Applied Biosystems, Foster, CA, USA). Standardization of the relative quantitation of expression levels of selective gene was carried out by real time RT-PCR (ABI Prism 7000 SDS, Applied Biosystems) with cDNA as template using TaqMan probe assay. Primers and probe for the AR (target gene) and TBP (endogenous control) were designed by Applied Biosystems. A singleplex reaction mix was prepared according to the manufacture's protocol of Assay-on-Demand gene expression products.

The mean expression level of *AR *gene was calculated for breast tissue normalized to a house keeping gene *TBP *(TATA box binding protein), an endogenous control. The average CT was calculated for both interest of gene (AR) and house keeping gene (TBP). The 2-ΔΔCT method was used to calculate relative changes in gene expression determined from real-time quantitative PCR experiments. The relative AR gene expression level was also normalized to a calibrator consisting of a pool of normal breast tissue specimens. For this, specimen of adjacent normal breast tissue from 12 of the breast cancer patients was used as a source of normal RNA. Final results, expressed as n-fold differences in AR gene expression relative to TBP gene and normal breast tissue (the calibrator), termed *n*AR, were determined.

The CAG repeat length was not examined in adjacent normal tissue since the amount of tissue was very less and with lots of fat (adipose tissue) hence; only RNA was isolated and used to calibrate AR gene expression of tumour tissue.

### Neo-adjuvant Chemotherapy

In 39 cases of locally advanced breast cancer (LABC) cases neo adjuvant chemotherapy (NACT) was given prior to surgery. For NACT, three cycles of FAC regime (cyclophosphamide 500 mg/m2, adriamycin 50 mg/m2, 5-fluorourail 500 mg/m2) were given at three weekly intervals and the patients were assessed both clinically and by ultrasound for response in the form of reduction in breast tumor size and axillary lymph node status. After 3 weeks of the last cycle of NACT, the patients were taken up for modified radical mastectomy, after a preoperative clinical and ultrasonological assessment to check for debulking of tumor. Clinical responders were defined as patients with a complete response i.e. more than 50% regression in maximum diameter of initial tumor after 3 cycles of NACT. Non-responders were patients with a minimal response i.e. less than 50% regression, no change or increase in tumor size [3,11,12]. Among 30 cases follow of drug response to NACT along with matched pre and post neoadjuvant chemotherapy biopsy samples were available. The main prognostic factors are presented in Table 1.

**Table 1:** Patient Characteristics (n = 30)

	No. of patients (%)
**Age**	
Mean	44.30
Range	26-65
	
**Menopausal Status**	
Premenopausal	13 (43.33)
Postmenopausal	17 (56.66)
	
**Tumor size before NACT**	
<5 cms	4 (13.33)
5-8 cms	17 (56.66)
8-10 cms	6 (20.00)
>=10 cms	3 (10.00)
	
**Tumor size after NACT**	
<5 cms	19 (63.33)
5-8 cms	8 (26.66)
8-10 cms	3 (10.00)
>=10 cms	0 (0)
	
**Lymph node status before NACT**	
N1	9 (30.00)
N2	19 (63.33)
N3	2 (6.66)
	
**Lymph node status after NACT**	
N0	15 (50.00)
N1	9 (30.00)
N2	4 (13.33)
N3	2 (6.66)
	
**Clinical response**	
Responders	19 (63.33)
Non-responders	11 (36.66)
	
**Her-2neu Status**	
Positive	11 (36.66)
Negative	19 (63.33)
	
**ER Status**	
Positive	14 (46.66)
Negative	16 (53.33)

### Statistical Analysis

For AR gene, allele lengths were compared between cases and controls. Comparisons were made for the mean allele length, and separately for the shorter and the longer alleles. Mann-Whitney U test was applied to test for the significant difference in CAG repeat length between cases and controls. Dichotomous categories for CAG repeats were generated at all possible cut-off points to assess the association with disease risk. These categories were analyzed using χ2/Fisher's exact tests for comparison. The statistical significance was considered for p ≤ 0.05. The univariate logistic regression analysis was performed by considering CAG repeat polymorphism (AR2 allele, ≥20 CAG repeats) as dependent variable and potential predictors as family history, a well known risk factor and stage, grade, estrogen-receptor (ER) and androgen-receptor status, the well known prognostic markers, by means of case-only analysis. The results under the logistic regression analysis were interpreted in terms of unadjusted and adjusted odds ratio and 95% CI for carrying AR2 allele and thereby the associated breast cancer susceptibility. The factors ER and AR status were not considered under multivariate analysis as data was available only for 51% cases. The association of AR2 alleles among cases and controls was analyzed in the matched form (McNemar's test) related to age of onset of disease however the same could not be performed on rest of the factors because the information on stage, grade ER and AR status was obtained on the surgical specimens which were available among cases only. Wilcoxon Signed Rank test was performed for comparing pre and post therapy AR mRNA expression levels among responders and non-responders. The SPSS (version 17.0) software was used to perform the analysis of the present data.

## Results

### AR-CAG repeats polymorphism

**Figure 1:**
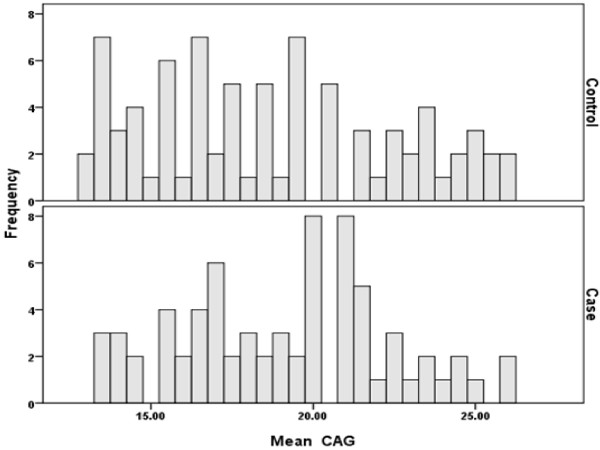
**Histogram of CAG repeat length among cases and controls**.

**Figure 2:**
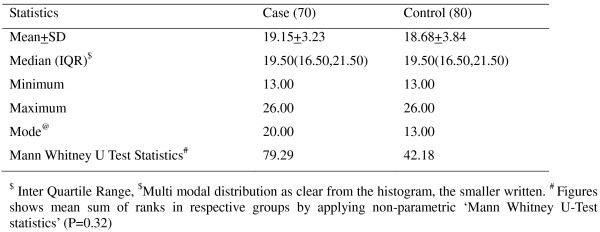
**Descriptive statistics of CAG repeat polymorphism in case and control**.

The assayed population showed 14 different CAG alleles, ranging from 13 to 26 repeats and the most frequent alleles were 19, and 20, in cases and 14 in controls. The frequency of the CAG repeat length showed bimodal distribution as clear from the histogram (Figures 1 and 2). For the study subject the size of the two AR alleles was determined. The mean AR allele size was 19.2 ± 3.2 units for cases and was 18.7 ± 3.8 units for controls. On average, the number of CAG repeats of the longer of the two AR alleles (the "long" allele) was 20.1 ± 3.5 for cases and was 19.2 ± 3.8 for controls. The mean length of the shorter of the two alleles (the "short" allele) was 18.2 ± 2.9 for cases and 18.2 ± 3.9 for controls. The average of CAG repeats was not significantly different between cases and controls, neither when the average of both CAG alleles of an individual was considered (P = 0.90) (on the presumption of random X inactivation of the AR gene in target tissues) nor when the short (P = 0.39) and long (P = 0.11) alleles were measured separately (Table 2). Since the mean of short and long alleles did not show differences between cases and controls and the same was true when the average of both CAGn was considered, the further analysis was done defining short allele as CAGn < 20 (AR1) and the long as CAGn ≥ 20 (AR2). This cut-off point was chosen because the mode of CAGn in cases and controls was approximately near to 20 repeats. Marginal significant difference was observed when women for whom the average of both CAG repeat alleles did not exceed 20 (CAGn < 20) compared with women having average of CAG repeats more than 20 (P = 0.09). However women carrying single long allele AR genotypes (AR1AR2) were at significantly higher risk of developing the disease compared with those bearing both short allele AR genotypes (AR1AR1) (P = 0.02) (Table 3) although, no trend in risk was observed considering AR2AR2 genotype. The odds ratio of carrying AR2 allele among breast cancer patients was found statistically insignificant on both matched (p = 0.230) (Table 4) and unmatched (p = 0.160) (Table 5) analysis in early-onset cases. Other factors modifying the risk for breast cancer such as family history, stage, grade, ER and AR status of disease were determined and the differences were assessed among cases only according to AR2 allele but these findings were not found statistically significant (Table 5).

**Table 2:** Association of *AR*-CAG polymorphism with breast cancer risk (N = 150)

Group	Cases (N = 70)	Controls (N = 80)	P value	OR
CAGna ≥ 20 repeats (AR2)	34 (*48.6%*)	28 (*35.0%*)	0.09	1.75

<20 repeats (AR1)	36 (*51.4%*)	52 (*65.0%*)	0.09	0.57

**Table 3:** Zygosity of exon 1 site of AR gene among breast cancer affected and Controls (N = 150)

Group	Cases (N = 70)	Controls (N = 80)	P value
**AR1AR1**	30 *(42.8%)*	45 *(56.2%)*	Referent
**AR1AR2**	15 *(21.4%)*	7 *(8.7%)*	0.02
**AR2AR2**	25 *(35.7%)*	28 *(35.0%)*	0.47

**Table 4:** Association of AR-CAG polymorphism with breast cancer (matched analysis)

		Control		OR (95% CI), p
		**AR2**	**AR1**	
			
**Case**	AR2	18	21	1.615 (0.772,3.511), 0.230
	AR1	13	35	

**Table 5:** Results of Binary Logistic Regression analysis of CAG (AR2) repeats in relation to covariates (n = 70)

Variables			Unadjusted		Adjusted	
	Distribution of Genotype in Cases		OR (95% C.I.)	P value	OR (95% C.I.)	P Value
	AR1 (36) (R%, C%)!	AR2 (34) (R%, C%)!				
**Age of onset**						
Late onset (20)	8 (40, 22.2)	22 (44, 64.8)	1.000	0.397	1.000	0.160
Early onset (50)	28 (56, 77.8)	12 (60, 35.2)	0.786 (0.450, 1.373)		0.454 (0.151, 1.365)	

**Family History**						
No FH (59)	30 (50.8, 83.3)	29 (49.2, 85.3)	1.000	0.763	1.000	0.523
Having FH (11)	6 (54.5, 16.7)	5 (45.5, 14.7)	0.833 (0.254, 2.731)		0.637 (0.160, 2.539)	

**Stages of Disease**						
Initial stages (I + II) (23)	13 (56.5, 36.1)	10 (43.5, 29.4)	1.000	0.884	1.000	0.486
Advance stages (III + IV) (47)	23 (48.9, 63.9)	24 (51.1, 70.6)	1.043 (0.589, 1.849)		1.461 (0.503, 4.243)	

**Grade**						
Lower (I + II) (52)	29 (55.8, 80.6)	23 (44.2, 67.6)	1.000	0.350	1.000	0.120
Higher (III) (18)	7 (38.9,19.4)	11 (61.1, 32.4)	1.571 (0.609, 4.054)		2.470 (0.781, 7.817)	

**Estrogen receptor status**	**N= 18**	**N= 18**	1.000	0.530		
Positive (10)	6 (60, 33.3)	4 (40, 22.2)	0.667 (0.188, 2.362)			
Negative (26)	12 (46.2, 66.7)	14 (53.8, 77.8)				

**Androgen receptor status**	**N = 20**	**N = 26**	1.000			
Positive (16)	8 (50, 40)	8 (50, 30.7)	0.668 (0.196, 2.263)	0.516		
Negative (30)	12 (40, 60)	18 (60, 69.3)				

!:(R%: row percentages, C%: column percentages)

### Correlation between AR mRNA Expression and (CAGn) Length Polymorphism

To investigate the influence of CAG repeat length polymorphism on AR mRNA level in breast cancer, total RNA from breast tumour samples were reversed transcribed to cDNAs and nAR values were estimated, simultaneously from the same samples which were used for the genotyping of CAG repeat length. To determine the cut-off point for altered AR expression in breast cancer tissue, the normal expression was an n-fold ranging from 0.55 to 1.80. Based on normal expression range, 14 tumors (35%) showed ARmRNA over expression and 20 tumors (50%) showed AR mRNA under expression. The assayed population showed 13 different CAG alleles, ranging from 14 to 26 repeats. For the association study the cut-off value was taken 20 CAG repeats and the data showed that 69.2% of the genotypes with 20 or more than 20 CAG repeats were down regulated for AR mRNA expression as compared to 30.7% with up-regulation whereas considering genotypes with less than 20 CAG repeats, there was no great difference in the frequency of two groups (Table 6).

**Table 6:** Association between AR mRNA expression and AR- CAG repeat length (n = 34)

Cases/Genotype	CAGna ≥ 20 repeats (N = 13)	CAGna < 20 repeats (N = 21)	p value
Up regulated for AR mRNA expression	4 *(30.7%)*	10 *(47.6%)*	0.47
Down regulated for AR mRNA expression	9 *(69.3%)*	11 *(52.4%)*	

### Response to chemotherapy and correlation with mRNA expression

Among 30 patients of locally advanced breast cancer in whom follow up for therapeutic response to NACT was available, clinical response was observed in 19 of 30 (63.4%) patients where as 11 (36.6%) patients were found non-responders. The mRNA expression level of AR estimated in matched pre and post chemotherapy tissue samples showed significant over expression of AR mRNA in responding patients (p < 0.02) compared to non-responders in pre therapy samples. The AR mRNA expression among responders get significantly reduced following chemotherapy (p = 0.014) while in non responders the AR mRNA expression was found increased in post therapy samples compared to pre therapy samples, however it was not found statistically significant (Table 7).

**Table 7:** Mean AR mRNA expression of response group (pre NACT vs. Post NACT) (n = 30)

Response group	Pre NACT (n = 30)	Post NACT (n = 30)	P value
Responder	16.39	1.95	0.014
Non responder	3.52	7.51	0.172
	P < 0.02	P < 0.09	

## Discussion

Steroid hormones are key factors in the development and growth of tumors in hormone dependent tissues especially breast. The action of steroids is mediated by steroid hormone receptors, which are members of nuclear receptor family of ligand-activated transcription factors. The role of androgens and androgen receptors in breast carcinogenesis is poorly understood, although wide spread expression of AR in breast cancer suggests that it may have significant biological and clinical relevance. Recently some studies have suggested an association of (Gln)n tract with differences in Breast cancer risk. The relationship has been examined in several case-control studies in different populations; some have related longer CAG repeats with an increase in breast cancer risk [13-15] whereas others have limited the impact of AR-CAG repeat on Breast Cancer [16-19]. Because AR is located on the X chromosome, breast epithelial cells in women express only one of the two AR alleles; the other is inactivated due to dosage compensation. The inability to distinguish between the active and inactive X allele of female case and control subjects was obviated by testing the risk differences between individuals with each allele (AR1/AR2) and grouping them into three risk categories (AR1AR1, AR1AR2 and AR2AR2). The frequent alleles found in the present study were 19 and 20 repeats (range; 13-26) in Breast cancer cases as reported in Quebec population [13], the most frequent CAG repeat allele being 21 repeats [13]. The range of CAG repeat length in our control population resembles those reported from other populations. The CAG repeats varied from 14 to 31 in Japanese [20], 19 to 27 in Philippines [15] and 14 to 30 in Tenerife population (Spain) [21]. The mean of short and long alleles between cases and controls in study population did not show any difference and the same was true when the average of both CAGn was considered as well as using a mean cut-off value of 20 repeat units, however women carrying single long allele AR genotypes (AR1AR2) were at significantly higher risk of developing the disease compared with those bearing short allele AR genotypes (AR1AR1) [3.21(1.19 - 8.60), P = 0.02]. In a population based case-control study of African-American women, although, overall significant association between CAG repeat polymorphism and breast cancer risk was not observed, among women with a first-degree family history of breast cancer, longer CAG repeats were associated with a significantly higher risk. Women carrying at least one longer allele (CAGn ≥ 22) had a 3-fold increased risk compared to those with two shorter alleles [22]. Elhaji and colleagues described a 2.4-fold increased risk of breast cancer associated with allele lengths of 26 CAG repeats or greater [23]. On similar lines Giguere et al. (2001) examined the inverse association of CAG repeat length on breast cancer risk in Quebec. They reported an OR of 0.5 for women with mean allele sizes of 20 CAG repeats or less. Women with short CAG alleles (39 repeats total from both alleles) have a 50% reduction in risk compared with women for whom the sum of repeats is 40 or more. Whereas in women of Greek decent, an association for breast cancer risk with short alleles (≤22 repeats) for the AR gene was observed [24]. Few studies have reported a slight, yet, not statistically significant increase in the risk of breast cancer associated with long CAG alleles [16,18,19]. Dunning et al. (1999) failed to observe a difference in susceptibility to breast cancer between women with 22 or less glutamine residues (*i.e.*, ≤21 (CAG) n repeats) when compared with those with at least one allele with 23 glutamine residues or more (*i.e.*, ≥22 (CAG)n repeats in Caucasian females from the East-Anglia region of the UK. Conflicting results in association studies may arise for several reasons including differences in ethnic (genetic) background, gene-gene or gene-environment interactions and limited sample size.

While assessing the impact of CAG repeat length on Androgen receptor mRNA expression in case subjects, it was observed that more than 60% of the genotypes with (≥20 CAG repeats) were down regulated for AR mRNA expression although statistical significance could not be established. However considering cases with (<20 CAG repeats) no great difference was found in the frequency of cases up regulated and down regulated for AR mRNA expression. Further 18 (69.3%) out of 26 cases with AR2 genotype showed negative nuclear AR immuno-histochemical staining. These results support the hypothesis that longer repeats might have reduced transactivation efficiency [25]. Interaction of AR protein is known to be dependent on tissue and promoter context and a decreased amount of AR protein (long CAGn) with low transcriptional activity in the cell would increase the breast cancer risk. Several studies have observed an association between increasing AR CAG repeat length and a linear decrease in AR transactivation activity (Choong et al.1996) [6,26-31]. Shorter alleles of the AR gene would be associated with a better response to circulating androgens, possibly resulting in better "repression" of breast cancer development and/or progression. However, the biological explanation for this observation is still uncertain. Comparing the mRNA expression level of AR gene in pre and post chemotherapy therapy patients showed that tumours of responders had the higher mRNA expression levels in pre NACT condition which got reduced after neoadjuvant chemotherapy and the difference was found to be statistically significant (p = 0.014). After neoadjuvant chemotherapy AR mRNA expression levels got reduced in tumors of responders, the reason could be, important cellular processes, e.g., DNA repair, apoptosis, which often occurs within 48 hours after chemotherapy exposure (Chang J 1999, Parton M 2002, Ellis PA 1998, Chang J 2000). According to one line of action, the translocation of Bax to mitochondria is one of the key steps for Bax-mediated apoptosis [32,33] and AR is required for UV to induce the translocation of endogenous Bax to mitochondria, prior to apoptosis. Inhibition of AR expression by AR siRNA also suppressed the translocation of exogenous HA-Bax, thereby inhibiting HABax-induced apoptosis in prostate cancer cells [34]. The AR may thus serve as an important independent predictor of response to NACT and may help in the tailoring of the regime to a particular patient. It is true that the present study has its own strength and limitations. The major limitation is small sample size. In the logistic regression analysis limited factors were considered and for majority of them the analysis was unmatched, since for these, information could be achieved only from surgical specimens. But even with these limitations, the present study makes a substantial endeavor in enriching our knowledge towards better understanding of androgen receptor gene polymorphism (CAGn) and breast cancer risk as well as its role as a predictive marker in the understudied population of north India. Moreover the present study, to our knowledge, is the first report on association of *AR *with breast cancer from India.

## Conclusions

To summarize, we could not find a continuous gradient of risk associated with AR alleles of different sizes with breast cancer in Indian women, although women carrying single long AR allele genotype (AR1AR2) are at higher risk for developing breast cancer than those having both short (AR1AR1) or long alleles (AR2AR2). On the other hand AR appears as a promising predictive biomarker for response to neoadjuvant chemotherapy in locally advanced breast cancer patients. Additional work is necessary to elucidate the specific mechanisms by which the androgens and AR influences breast cancer cells proliferation and apoptosis. Although the role of AR as a potential new target for hormone therapy is recommended and it may serve as clinically useful predictor to therapy, the impact of AR in breast cancer needs further study, especially its association with growth factors. Once established, these may prove to be a useful target for planning therapeutic strategies for the treatment of breast cancer patients in future.

## Competing interests

The authors declare that they have no competing interests.

## Authors' contributions

C, PK, AC, LCS, AKM, DB and SS contributed to the designing of study and preparation of manuscript. All authors read and approved the final manuscript.
